# Functional brain networks assessed with surface electroencephalography for predicting motor recovery in a neural guided intervention for chronic stroke

**DOI:** 10.1093/braincomms/fcab214

**Published:** 2021-09-25

**Authors:** Rui Sun, Wan-Wa Wong, Jing Wang, Xin Wang, Raymond K Y Tong

**Affiliations:** 1 The Laboratory of Neuroscience for Education, Faculty of Education, the University of Hong Kong, Pokfulam, Hong Kong, China; 2 Department of Psychiatry and Biobehavioral Sciences, David Geffen School of Medicine, University of California Los Angeles, Los Angeles, CA, USA; 3 School of Mechanical Engineering, Xi’an Jiaotong University, Shaanxi, China; 4 Department of Biomedical Engineering, The Chinese University of Hong Kong, Shatin, Hong Kong, China

**Keywords:** neural guided intervention, stroke, functional connectivity, predictive biomarker, EEG

## Abstract

Predicting whether a chronic stroke patient is likely to benefit from a specific intervention can help patients establish reasonable expectations. It also provides the basis for candidates selecting for the intervention. Recent convergent evidence supports the value of network-based approach for understanding the relationship between dysfunctional neural activity and motor deficits after stroke. In this study, we applied resting-state brain connectivity networks to investigate intervention-specific predictive biomarkers of motor improvement in 22 chronic stroke participants who received either combined action observation with EEG-guided robot-hand training (Neural Guided-Action Observation Group, *n* = 12, age: 34–68 years) or robot-hand training without action observation and EEG guidance (non-Neural Guided-text group, *n* = 10, age: 42–57 years). The robot hand in Neural Guided-Action Observation training was activated only when significant mu suppression (8–12 Hz) was detected from participant’s EEG signals in ipsilesional hemisphere while it was randomly activated in non-Neural Guided-text training. Only the Neural Guided-Action Observation group showed a significant long-term improvement in their upper-limb motor functions (*P* < 0.5). In contrast, no significant training effect on the paretic motor functions was found in the non-Neural Guided-text group (*P* > 0.5). The results of brain connectivity estimated via EEG coherence showed that the pre-training interhemispheric connectivity of delta, theta, alpha and contralesional connectivity of beta were motor improvement related in the Neural Guided-Action Observation group. They can not only differentiate participants with good and poor recovery (interhemispheric delta: *P* = 0.047, Hedges’ *g* = 1.409; interhemispheric theta: *P* = 0.046, Hedges’ *g* = 1.333; interhemispheric alpha: *P* = 0.038, Hedges’ *g* = 1.536; contralesional beta: *P* = 0.027, Hedges’ *g* = 1.613) but also significantly correlated with post-training intervention gains (interhemispheric delta: *r* = −0.901, *P* < 0.05; interhemispheric theta: *r* = −0.702, *P* < 0.05; interhemispheric alpha: *r* = −0.641, *P* < 0.05; contralesional beta: *r* = −0.729, *P* < 0.05). In contrast, no EEG coherence was significantly correlated with intervention gains in the non-Neural Guided-text group (all Ps>0.05). Partial least square regression showed that the combination of pre-training interhemispheric and contralesional local connectivity could precisely predict intervention gains in the Neural Guided-Action Observation group with a strong correlation between predicted and observed intervention gains (*r* = 0.82r=0.82) and between predicted and observed intervention outcomes (*r* = 0.90r=0.90). In summary, EEG-based resting-state brain connectivity networks may serve clinical decision-making by offering an approach to predicting Neural Guided-Action Observation training-induced motor improvement.

## Teaser

Sun et al. report brain connectivity at resting state as a biomarker for predicting intervention gains of chronic stroke patients. Results show a strong correlation between predicted and observed intervention gains after receiving robot-assisted training combined with neural guided strategy. The findings have clinical potential in candidates selecting for the specific intervention.

## Graphical Abstract

**Graphical Abstract:**
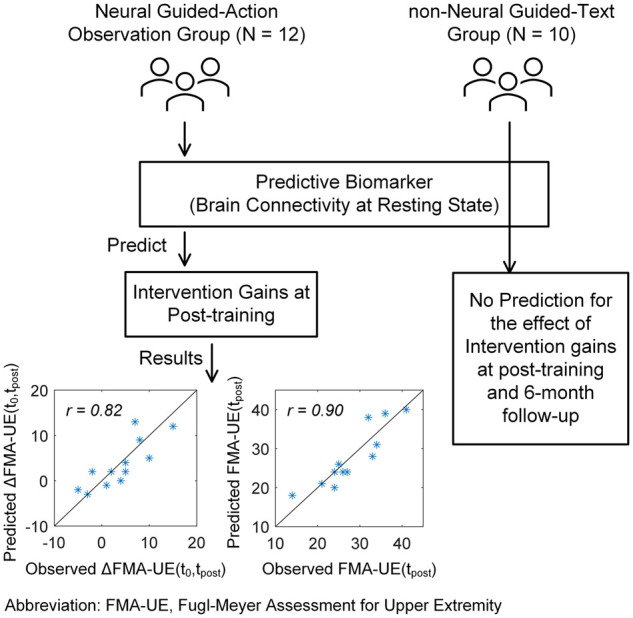

## Introduction

Stroke is one of the leading causes of long-term disability in the United States, especially in the elderly population, in which stroke incidence is highest. Of the 795 000 new sufferers of stroke, 26% remain disabled in necessary daily living activities, and 50% have reduced mobility due to hemiparesis.^1^^,^^2^ Effective rehabilitation strategies can improve the quality of daily life and help them regain their independence and return to society, which reduces the burden on themselves, their families and society. In chronic stroke rehabilitation, different types of intensive intervention training have been validated for their clinical benefits at the group level in our previous research.^3–5^ However, the patients’ response to an intervention is highly subject-specific at the individual level.^6^ So, is there a biomarker that can predict the intervention-induced motor improvement before rehabilitation training? The answer to this question can provide a basis for selecting candidates who are more likely to benefit from a specific intervention. Besides, making accurate predictions of rehabilitation gain could allow clinical teams, patients and families to establish reasonable expectation, optimize rehabilitation plan with realistic goals and appropriately allocate time and resources.^7^

Regarding stroke prognosis, clinical measurements^8–14^ can be used to explain long-term motor impairment outcomes. However, they are less likely to explain functional outcomes because these outcomes can be improved by movement strategies that compensate for motor impairments. Furthermore, although some patients with severe initial motor impairments have a proportional recovery, others do not in which clinical measurement cannot reliably discriminate.^7^ Currently, interest in biomarkers, including neurophysiological and neuroimaging markers, for predicting motor recovery and motor outcomes in clinical research is growing. Among these biomarkers, EEG reflects brain activity from the perspective of electrophysiology, and it is a low cost, high safety and convenient tool with a high temporal resolution to monitor neurological activity. EEG has been applied to the prognosis of motor impairment and recovery in acute^15^^,^^16^ and subacute^17–19^ stroke.

Convergent evidence supports the network-based approach for understanding the relationship between dysfunctional neural activity and motor deficits after stroke.^20–36^ EEG coherence is a mathematical method used to determine whether two or more sensors or brain regions have similar neuronal oscillatory activity.^37^ It can investigate functional brain connectivity and describe brain networks based on this connectivity. Wu et al.^36^ found that EEG coherence between ipsilesional primary motor cortex (M1) and ipsilesional premotor cortex (PM) was strongly related to motor deficits and improvements with virtual reality- and computer game-assisted recovery after stroke. The analysis was conducted on beta coherence between a seed region over the ipsilesional M1 and other brain regions. Information transmission in the brain occurs through a complex network instead of a single pathway. After stroke occurs, the brain networks responsible for transmitting information will be modulated. Some of the pathways in the brain network are disconnected or weakened due to neuronal and fibre cell death, while some of the pathways are enhanced due to weakened inhibitory function, which varies from person to person. Systematically defining what kind of brain connectivity network pattern is linked to a good recovery not only provides us with a biomarker for predicting intervention-induced motor improvement of chronic stroke patients in clinical trials, it also serves as an approach to understanding the neurological mechanisms of chronic stroke rehabilitation. In this study, brain connectivity was estimated via EEG coherence between electrodes overlying the motor and motor connected regions,^38^ including the M1, PM, somatosensory cortex (SI) and supplementary motor area (SMA). Brain connectivity was used to predict intervention-induced motor improvement with robot-assisted training combined with a neural guided strategy.

Compared to prognostic biomarkers, which provide information about the natural course of a disease, an intervention-specific predictive biomarker in chronic stroke predicts a patient’s response to treatment. It has significant potential for selecting the most appropriate participants for clinical training by predicting whether a patient is likely to benefit from a specific intervention in a clinical trial. For example, Mane et al.^6^ investigated intervention-specific predictive biomarkers of motor function improvements using EEG features in chronic stroke patients following two different upper-extremity rehabilitative interventions. Trujillo et al.^39^ assessed the relationship between resting EEG measures and motor outcomes in chronic stroke patients who underwent a robot-assisted rehabilitation programme to evaluate the utility of EEG to predict motor recovery. Here, we propose the existence of the intervention-specific predictive biomarkers for a robot-assisted training combined with a neural guided strategy. We hypothesize that brain connectivity at pre-training can predict the intervention gain of the robot-assisted training combined with a neural guided strategy. Furthermore, since the mechanisms of neuronal recovery elicited by different interventions are not identical, we hypothesize that the pre-training brain connectivity only uniquely can predict intervention gain in the robot-assisted training while it cannot be applied to control training. Investigating these intervention-specific predictive biomarkers can be further pursued to predict the expected response of the given interventions for chronic stroke patients. The patients with high predicted gains may then recommended being recruited. This research also provides systematic insight into the mechanisms of using EEG for predicting intervention-induced motor improvement.

## Materials and methods

### Participants

Twenty-four chronic stroke participants (age 34–68 years; 20 males/4 females) were recruited from the local community, as shown in Table 1. The inclusion criteria were as follows: (i) sufficient cognition to follow experimental instructions with Mini-Mental State Examination (MMSE) score > 21; (ii) moderate to severe motor impairments of the paretic upper limb [Fugl-Meyer Assessment for Upper Extremity (FMA-UE) < 47]^40^^,^^41^; and (iii) hemiparesis resulting from a single unilateral brain lesion with stroke onset more than 6 months before data collection. The exclusion criteria were as follows: (i) severe hand spasticity (spasticity during extension of the finger joints was more than 3 as assessed by the Modified Ashworth Scale)^42^; (ii) open hand wound or hand deformity; (iii) visual field deficits; (iv) aphasia, neglect and apraxia; (v) participation in any therapeutic treatment (‘outside therapy’) performed with the affected upper limb during the course of the study; (vi) history of alcohol, drug abuse or epilepsy; and (vii) bilateral infarcts, uncontrolled medical problems and severe cognitive deficits. All participants signed written informed consent according to the Declaration of Helsinki. The Joint Chinese University of Hong Kong-New Territories East Cluster Clinical Research Ethics Committee (CUHK-NTEC CREC) approved the experimental protocol (agreement #2014.705-T). This study was registered at www.clinicaltrials.gov, with the study identifier NCT02323061. Participants were screened by excluding the abnormal differences between post-training and 6-month follow-up FMA-UE (if it is 1.5 times the interquartile range larger than the third quartile or 1.5 times the interquartile range smaller than the first quartile) which may cause by violating the exclusion criteria (5). Two outlier participants (S13 and S22 in Table 1) were excluded.

**Table 1:** Demographics and clinical characteristics of the participants

Sub	GP	Gender	Age	Type	IH	TSS (yrs)	FMA-UE			Recovery condition	Training intensity	Lesion
							t_0_	t_post_	t_6m_			Location
S1	NG-AO	M	47	Hemo	L	2	20	24	26	good	1669	No MRI
S2	NG-AO	M	65	Isch	R	5	23	33	33^a^	good	1306	No MRI
S3	NG-AO	M	48	Hemo	R	1	17	25	25	good	1506	ITG, MTG, STG, MOG, angular, supramarginal
S4	NG-AO	M	68	Hemo	L	8	22	27	32	good	1453	Insula, putamen, IFG, temporal pole
S5	NG-AO	M	60	Isch	R	3	16	14	18	poor	1447	Insula, putamen, rolandic operculum, IFG
S6	NG-AO	M	61	Isch	L	11	22	24	24	poor	1657	PLIC, putamen
S7	NG-AO	F	68	Isch	R	2	25	26	26	poor	1380	No MRI
S8	NG-AO	F	48	Isch	R	1	36	41	48	good	1560	Putamen, insula
S9	NG-AO	M	53	Isch	L	1	41	36	40	poor	1289	MFG, precentral, IFG, postcentral, insula, SFG
S10	NG-AO	M	49	Isch	R	1	19	34	28	good	1382	MFG, SFG, precentral, supramarginal, SMA
S11	NG-AO	M	34	Isch	R	2	25	32	32^a^	good	1306	No MRI
S12	EXCLUDED											
S13	NG-AO	M	59	Isch, mild hemo	R	11	24	21	22	poor	1489	Brainstem
S14	nNG-text	M	42	Hemo	R	3	17	20	20	poor	1600	Insula, MTG, STG, putamen, temporal pole, rolandic operculum
S15	nNG-text	M	57	Hemo	L	5	28	33	24	good	1600	Insula, IFG, putamen
S16	nNG-text	F	52	Hemo	L	3	34	34	37	poor	1600	Insula, rolandic operculum, putamen
S17	nNG-text	M	48	Hemo	R	1	34	37	35	poor	1600	Insula, putamen
S18	nNG-text	M	50	Isch	L	1	24	22	22	poor	1600	Putamen, caudate nucleus
S19	nNG-text	M	57	Isch	R	6	13	23	20	good	1600	Insula, rolandic operculum, IFG
S20	nNG-text	M	50	Hemo	R	5	15	17	16	poor	1600	Insula, rolandic operculum, IFG, STG, putamen, temporal pole
S21	nNG-text	M	51	Hemo	L	2	20	19	28	good	1600	No MRI
S22	EXCLUDED											
S23	nNG-text	F	59	Isch	L	4	31	39	35	good	1600	No MRI
S24	nNG-text	M	57	Isch	R	7	20	25	21	good	1600	Insula, IFG, putamen, rolandic operculum, temporal pole

F, female; FMA-UE, Fugl-Meyer Assessment-Upper Extremity (maximum: 66); FG, inferior frontal gyrus; GP, group; Hemo, haemorrhagic; Isch, ischaemic; IH, injured hemisphere; IOG, inferior occipital gyrus; ITG, inferior temporal gyrus; L, left; M, male; MFG, middle frontal gyrus; MOG, middle occipital gyrus; MTG, middle temporal gyrus; PLIC, posterior limb of the internal capsule; R, right; SFG, superior frontal gyrus; SMA, supplementary motor area; STG, superior temporal gyrus; TSS, time since stroke; yrs, years.

a Missing data inferred by last observation.

### Intervention

**Figure 1:**
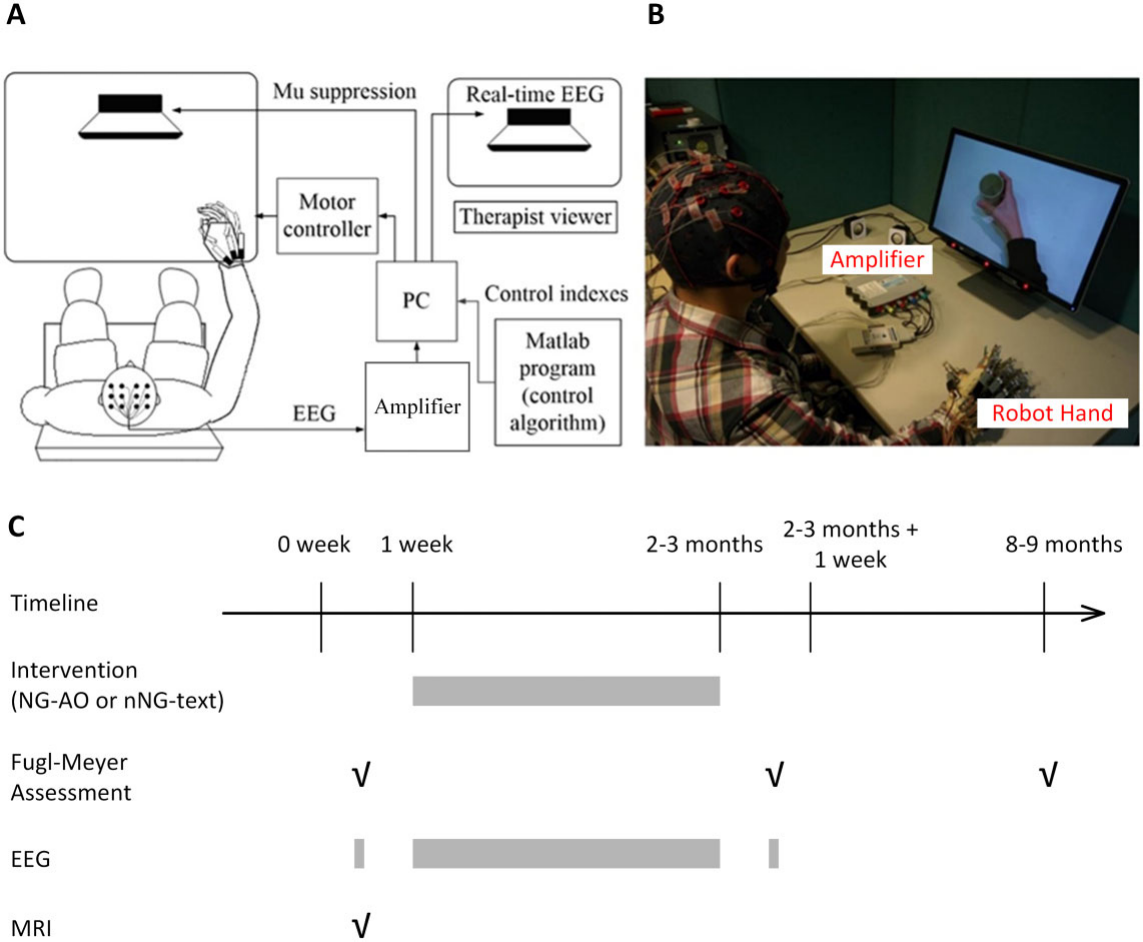
**Illustration of the intervention setup.** (**A**) An overview of the BCI-based neural guided training platform. (**B**) A photo taken in a real hand training session. (**C**) The experimental timeline shows that the intervention training started from the second week and lasted for 2 or 3 months. √ marks the timepoint of collecting FMA-UE scores, EEG data and MRI data.

The training was completed at the Biomedical Engineering Laboratory of The Chinese University of Hong Kong, Biomedical Engineering Laboratory of The Hong Kong Polytechnic University and the Chow Yuk Ho Technology Centre for Innovative Medicine. Stroke participants were required to come to the laboratory more than once before participating in the project to be familiar with the experimental environment and data collection procedure. The experimenter explained the purpose and process of the experiment, taught participants how to perform the motor imagery and motor observation tasks and answered the participants’ questions until they understood the design of the whole experiment. All participants received 20 sessions of robot-assisted hand training (Fig. 1A and B) with an intensity of 3–5 sessions per week that was completed within 5–7 weeks. The detailed structural information of the brain–computer interface (BCI)-based neural guided experimental platform can be found in the Supplementary material and in Sun et al.^5^ The participants were randomly assigned to one of two groups: (i) Neural Guided-Action Observation Group (NG-AO group): Action observation and motor imagery during playback of video of hand open/grasp with real-time EEG guidance to trigger the robot hand. (ii) non-Neural Guided-text group (nNG-text group): Motor imagery during display of text instruction of movement without EEG guidance, and the robot hand was randomly triggered. Each session of both groups was completed within 1.5 h. The details of the intervention procedure can be found in the Supplementary material and Wang et al.^43^

The FMA-UE (range: 0–66) was used to assess the improvement in motor performance at three time points (Fig. 1C): (i) FMA-UE(t_0_): data collected in the week before the intervention start date; (ii) FMA-UE(t_post_): data collected the week after finishing the intervention; and (iii) FMA-UE (t_6M_): data collected at the 6-month follow-up after the intervention. The intervention-induced motor gain was calculated as the difference in FMA-UE scores between t_0_ and t_post_, i.e. ΔFMA-UE(t_0_, t_post_), and as the difference between t_0_ and t_6M_, i.e. ΔFMA-UE(t_0_, t_6M_). FMA-UE was conducted by trained clinical assessors who were blinded to the experiment.

### EEG acquisition and MRI

Three minutes of awake, eyes-open, resting-state brain activity was captured by surface EEG using active electrodes (g.LADYbird, g. Tec Medical Engineering GmbH, Austria) and an amplifier (g.USBamp, g. Tec Medical Engineering GmbH, Austria) at the periods of one week before and one week after the intervention as well as at the beginning of each training session (Fig. 1C). Therefore, twenty-two sets of EEG data were recorded in total for each participant. Sixteen active electrodes were placed over the motor and motor connected regions in the central area according to the international 10–20 system (C1, C2, C3, C4, C5, C6, Cz, FC1, FC2, FC3, FC4, FCz, CP1, CP2, CP3 and CP4). EEG signals were referenced to a unilateral earlobe, grounded at a frontal position (Fpz), and sampled at 256 Hz. EEG signals were also processed in real-time using a bandpass filter (2–60 Hz) and a notch filter (48–52 Hz) to remove artefacts and power line interference, respectively. All electrodes were appropriately filled with a conductive gel to ensure that the transmission impedance remained below 1 kOhm.

Sixteen subjects who had no MRI contraindications (e.g. metallic implants, claustrophobia, pacemakers or unwilling to do MRI scan) had MRI scans at one week before the intervention (Fig. 1C), with eight subjects in each group. A 3 T Philips MR scanner (Achieva TX, Philips Medical System, Best, Netherlands) with an 8-channel head coil was used to acquire high-resolution T_1_-weighted anatomical images [repetition time (TR)/echo time (TE) = 7.47/3.45 ms, flip angle = 8°, 308 slices, voxel size = 0.6 × 1.042 × 1.042 mm^3^] using a T_1_-turbo field echo (TFE) sequence (ultrafast spoiled gradient echo pulse sequence). We used MRI imaging to find the lesion location (see Table 1), which should be provided because it affected EEG data due to the lesion.

### Coherence

Functional connectivity between brain regions was estimated from EEG coherence between electrodes overlying the corresponding regions.^38^ Coherence is one mathematical method used to determine if two or more sensors, or brain regions, have similar neuronal oscillatory activity.^37^ Coherence ranges from zero to one, with a value near one indicating that EEG signals have similar phase and amplitude differences at all time points and a value near zero indicating that signals have a random difference in phase and amplitude.^37^ The EEG coherence calculation details can be found in the Supplementary material. In this study, mean coherences in four frequency bands, delta (1–4 Hz), theta (4–8 Hz), alpha (8–14 Hz) and beta (14–30 Hz), were calculated for each pair of eight electrodes (C3, C4, FC3, FC4, CP3, CP4, FCz and Cz), which overlying areas responsible for the planning, control and execution of voluntary movements. The primary motor area was defined as either C4 or C3 (ipsilesional or contralesional M1), which control voluntary movements. The SMA was defined as Cz and associated with the function of cortical organization of movement. The premotor area was defined as either FC4 or FC3 (ipsilesional or contralesional PM), which plays a role in planning movement, in the spatial guidance of movement and in the sensory guidance of movement. The somatosensory area was defined as either CP4 or CP3 (ipsilesional or contralesional SI), which receives and processes sensory information from the entire body.^44^ The functional connectivity can be summarized as interhemispheric connectivity (**InterHemi:** C3-C4, C3-FC4, C3-CP4, FC3-C4, FC3-FC4, FC3-CP4, CP3-C4, CP3-FC4 and CP3-CP4), ipsilesional local connectivity (**IpsiLHemi**: C3-FC3, C3-CP3 and FC3-CP3), contralesional local connectivity (**ContraLHemi**: C4-FC4, C4-CP4 and FC4-CP4), ipsilesional to SMA connectivity (**IpsiL-SMA**: C3-Cz, FC3-Cz and CP3-Cz) and contralesional to SMA connectivity (**ContraL-SMA:** C4-Cz, FC4-Cz and CP4-Cz). Electrode arrays from individuals with infarcts in the left hemisphere were flipped across the midline for subsequent analyses.

### Statistical analysis

Statistical analysis was performed using IBM SPSS 22 software (SPSS Inc., Chicago, IL, USA). The missing data (6-month follow-up FMA-UE for 2 out of 22 participants) were inferred by the last observation (post FMA-UE) carried forward. Statistical analysis of the outcome measure, including FMA-UE (each item is scored on a 3-point ordinal scale), was conducted using the non-parametric tests while outcome measure of EEG coherence was conducted using the parametric tests. The Friedman test was applied to verify the statistical significance of changes between FMA-UE(t_0_), FMA-UE(t_post_), and FMA-UE(t_6M_) for each group separately. The Wilcoxon signed-rank test was used as a *post hoc* test to examine significant changes in different combinations of the three time points for FMA-UE scores.

In each of the NG-AO and nNG-text group, the participants were categorized as having good recovery [whose ΔFMA-UE(t_0_, t_post_) or ΔFMA-UE(t_0_, t_6M_) exceed minimal clinically important difference (MCID) which is 4 for FMA-UE] or poor recovery (remaining participants) as shown in Table 1 eleventh column. Permutation *t*-tests were applied to compare InterHemi, IpsiLHemi, ContraLHemi, IpsiL-SMA and ContraL-SMA connectivity between the participants with good recovery and with poor recovery. In permutation test, all possible combinations are considered. False Discovery Rate (FDR) was used deal with the multiple comparison correction, which adjusts *P*-values in a way that controls the family-wise error rate.^45^

Spearman correlation analysis was used to investigate the correlations between resting EEG coherence of each electrode pair at pre-training (t_0_) and intervention gains at post-training [FMA-UE(t_0_, t_post_)] and 6-month follow-up [FMA-UE(t_0_, t_6M_)] for each group separately. Permutation testing was used to further validate the significant results generated by Spearman correlations. Changes in FMA-UE scores [FMA-UE(t_0_, t_post_), FMA-UE(t_0_, t_6M_)] were randomly shuffled 5000 times to obtain a null distribution and the correlation coefficient of each arrangement was recalculated. The calculated *P*-values represent a distribution of the null hypothesis that there is no relationship between the two variables. This procedure provides a robust estimation of statistical significance reducing the Type-I errors, at the same time preserves the power of the study limiting Type-II errors.^6^ Finally, assuming Spearman’s rank correlation coefficient of 0.6, the sample size of both NG-AO group (*N* = 12) and nNG-text group (*N* = 10) satisfy the minimum requirement (*N* ≥ 9) to achieve a statistical power of 80% with a significance level of *α* = 0.05.^6^

To investigate the effect of the functional connectivity network on predicting intervention gains in the participants receiving the neural guided intervention, partial least squares (PLS) regression^46^ was applied to investigate the fundamental relationship between EEG coherence at pre-training (t_0_) and changes in FMA-UE at the post-training [FMA-UE(t_0_, t_post_)] and 6-month follow-up [FMA-UE(t_0_, t_6M_)] in the NG-AO group. The inputs of PLS model (pre-training EEG coherences) should not only have a strong correlation with intervention gains but also be able to discriminate good and poor recovery. PLS regression is particularly suitable when the matrix of predictors (number of EEG coherences = 20) has more variables than observations (sample size = 12) and when there is multicollinearity among predictors. The significance level for all statistical analyses was set at *P* < 0.05.

### Data availability statement

The EEG and MRI data that support the findings of this study are available on request from the corresponding author for the research purposes. The data are not publicly available due to their containing information that could compromise the privacy of research participants.

## Results

### Participants

The demographics and clinical characteristics of the participants in both groups are shown in Table 1. All participants completed the target number of training sessions. The training intensity was 1436.83 ± 159.94 repetitions in NG-AO group while 1600 repetitions in nNG-text group in which the ‘success rate’ of triggering the robot hand was set as 80% (see Supplementary material: Intervention Procedure). No significant difference was observed between the NG-AO and nNG-text groups in terms of age (*P* = 0.226), stroke onset time (*P* = 0.856), and FMA-UE(t_0_) (*P* = 0.724). Besides, training intensity, age, stroke onset time, FMA-UE(t_0_) shows no significant correlation with the clinical motor improvements at the post-training [ΔFMA-UE(t_0_, t_post_)] and 6-month follow-up [ΔFMA-UE(t_0_, t_6M_)] in each group separately (Ps>0.05).

### Clinical outcomes

**Figure 2:**
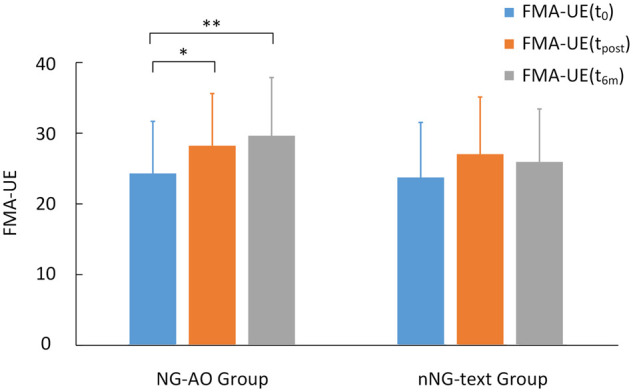
**Two groups of FMA-UE scores (mean ± standard deviation) from the pre-training, post-training and 6-month follow-up assessments.** The scores in the NG-AO group showed significant gains $\left[\chi^{2}\left(2\right)=8.512,\;P=0.014\right]$ in upper-extremity motor function at both the post-training (Z=-2.004, P=0.045) and 6-month follow-up (Z=-2.634, P=0.008) assessments, while the scores in the nNG-text group showed no significant gains $[\chi^{2}\left(2\right)=5.568,\;P=0.062$]. * indicates *P* < 0.05 and ** indicates *P* < 0.01.

In the NG-AO group, the mean FMA-UE scores significantly differed between each time point [$\chi^{2}\left(2\right)=8.512,\;P=0.014]$, as shown in Fig. 2. *Post hoc* analysis with Wilcoxon signed-rank tests was conducted with a Bonferroni correction applied, resulting in significant improvements in FMA-UE scores at the post-training (Z=-2.004, P=0.045) and at the 6-month follow-up (Z=-2.634, P=0.008). There was no significant difference in FMA-UE scores between the post-training and 6-month follow-up assessments (Z=-1.355, P=0.176). These results indicate long-term sustainable upper-limb functional recovery of participants in NG-AO group where neural guided strategy was applied. In the nNG-text group, there was no significant intervention effect on FMA-UE scores across the pre-training, post-training and 6-month follow-up assessments [$\chi^{2}\left(2\right)=5.568,\;P=0.062]$. In each group separately, the clinical motor improvements at the post-training [ΔFMA-UE(t_0_, t_post_)] and 6-month follow-up [ΔFMA-UE(t_0_, t_6M_)] assessments showed no significant correlations with the pre-training FMA-UE scores (all Ps>0.05), indicating no predictive effect of baseline FMA-UE score for intervention gains.

### Brain functional connectivity

**Figure 3:**
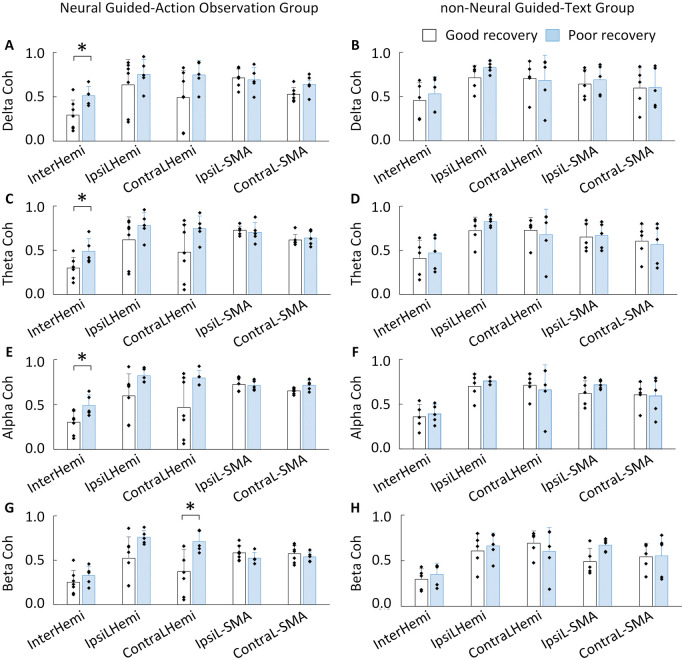
**Characterizing participants with good and poor recovery by pre-training EEG coherence of four frequency ranges (delta, theta, alpha and beta) and five brain connectivity networks (interhemispheric, ipsilesional local, contralesional local, ipsilesional-SMA and contralesional-SMA) in two groups.** (**ACEG**) Interhemispheric connectivity (delta, theta and alpha) and contralesional connectivity (beta) at pre-training can significantly differentiate participants with good (*N* = 7) and poor recovery (*N* = 5) in the NG-AO group. (**BDFH**) No brain connectivity showed a significant difference between participants with good (*N* = 5) and poor recovery (*N* = 5) in the nNG-text group. * indicates *P* < 0.05.

In the NG-AO group, the InterHemi of delta, theta and alpha was significantly different between the participants with good recovery and the participants with poor recovery (Fig. 3A, C and E; delta: *P* = 0.047, Hedges’ *g* = 1.409; theta: *P* = 0.046, Hedges’ *g* = 1.333; alpha: *P* = 0.038, Hedges’ *g* = 1.536). Contralesional local connectivity of beta was significantly different between participants with good recovery and poor recovery (Fig. 3G; beta: *P* = 0.027, Hedges’ *g* = 1.613). There were no significant differences between participants with good and poor recovery on EEG coherences for the remaining electrode pairs (Ps>0.05). In the nNG-text group, there was no EEG coherence that significantly differentiated participants with good and poor recovery (Fig. 3B, D, F and H; all Ps>0.05).

**Figure 4:**
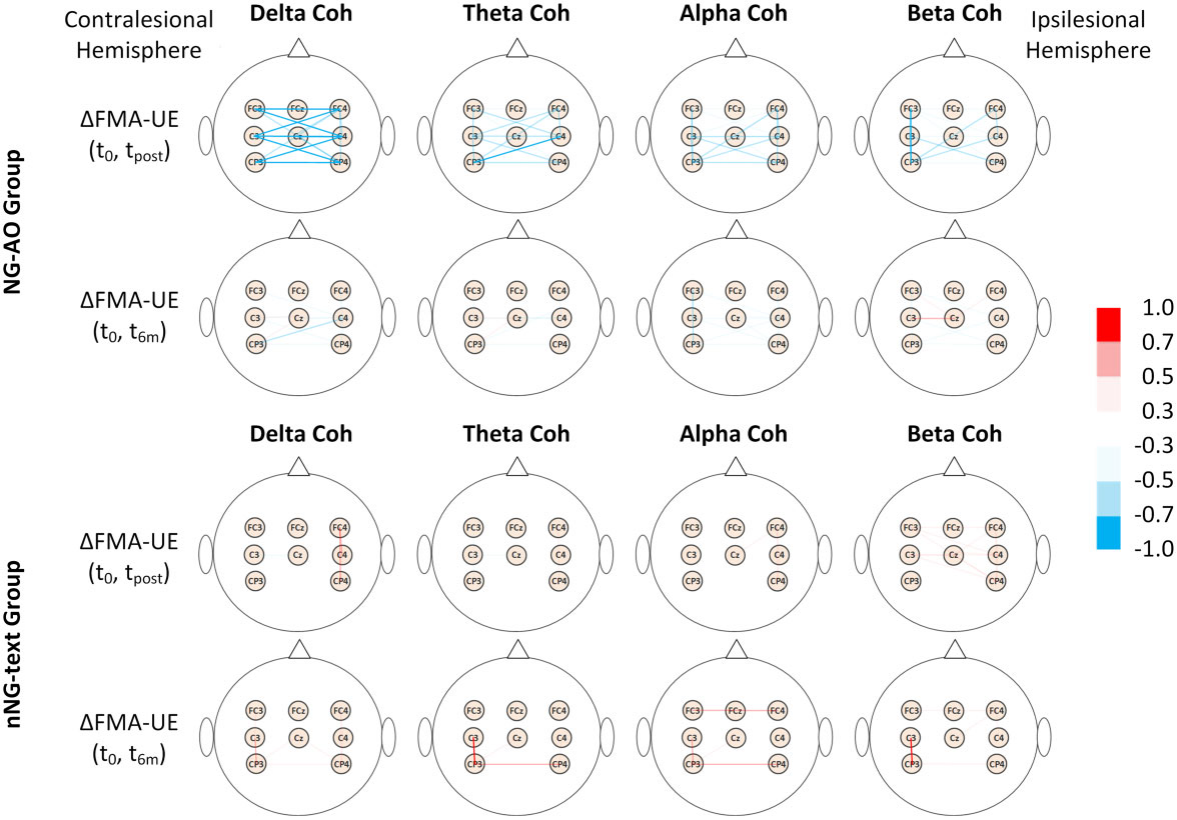
**Coherence network map associated with intervention gains in the two groups.** The colour of the lines indicates the correlation coefficient between EEG coherences in delta, theta, alpha, and beta and intervention gains at the post-training and 6-month follow-up assessments.

**Figure 5:**
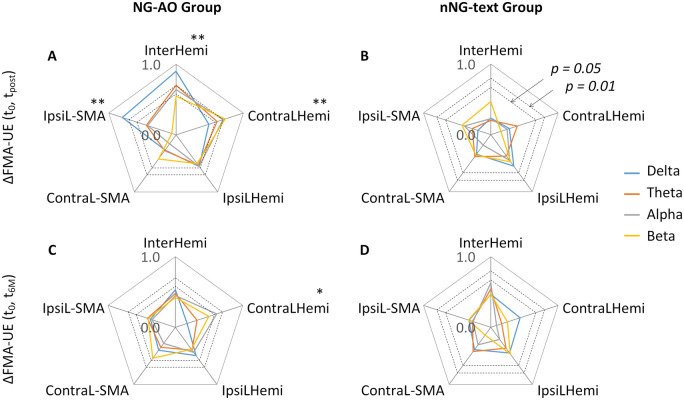
**Brain connectivity at pre-training associated with intervention gains in the two groups.** (**A**) Interhemispheric, contralesional local and ipsilesional-SMA connectivity at pre-training were significantly correlated with intervention gains at the post-training assessment in the NG-AO group (all *P*s < 0.01). (**B**) No brain connectivity had a significant correlation with intervention gains at the post-training assessment in the nNG-text group (*N* = 12; all *P*s > 0.05). (**C**) Contralesional local connectivity at pre-training was significantly correlated with intervention gains at the 6-month follow-up in the NG-AO group (*N* = 10; *P* < 0.05). (**D**) No brain connectivity was significantly correlated with intervention gains at the 6-month follow-up assessment in the nNG-text group (all *P*s > 0.05). * indicates *P* < 0.05 and ** indicates *P* < 0.01.

Figure 4 demonstrates the correlations between EEG coherence for each electrode pair and motor gains at the post-training and 6-month follow-up assessments [ΔFMA-UE(t_0_, t_post_), ΔFMA-UE(t_0_, t_6M_)]. The colour of the line linking each electrode pair is tuned by the correlation coefficient. Supplementary Tables 1–4 summarize the correlation coefficients between EEG coherences of delta, theta, alpha, and beta and ΔFMA-UE(t_0_, t_post_) and ΔFMA-UE(t_0_, t_6M_) in both the NG-AO and nNG-text groups. For the nNG-text group, no EEG coherence in any frequency band had a significant correlation with ΔFMA-UE(t_0_, t_post_) or ΔFMA-UE(t_0_, t_6M_) (Fig. 5B and D; all *P*s > 0.05). For the NG-AO group, InterHemi of delta (C3-C4, C3-FC4, C3-CP4, C4-FC3, C4-CP3, FC3-FC4, FC3-CP4, FC4-CP3 and CP3-CP4, all *P* < 0.05; Supplementary Table 1), theta (C3-C4, C3-CP4, C4-CP3, FC4-CP3 and CP3-CP4, all *P* < 0.05; Supplementary Table 2), and alpha (C3-CP4, C4-CP3 and CP3-CP4, all *P* < 0.05; Supplementary Table 3), contralesional local connectivity of theta (C3-CP3, *P* < 0.05; Supplementary Table 2), alpha (C3-FC3, C3-CP3 and FC3-CP3, all *P* < 0.05; Supplementary Table 3), and beta (C3-FC3, C3-CP3, FC3-CP3, all *P* < 0.05; Supplementary Table 4), and ipsilesional-SMA connectivity of delta (C4-Cz, *P* < 0.05; Supplementary Table 1) have significant correlations with ΔFMA-UE(t_0_, t_post_) (Fig. 5A). Contralesional local connectivity of alpha (FC3-CP3, *P* < 0.05, in Supplementary Table 3) has a significant correlation with ΔFMA-UE(t_0_, t_6M_) (Fig. 5C), indicating its potential for predicting long-term motor improvement.

Combining the results of the permutation *t*-tests and correlation analyses, the resting EEG InterHemi of delta, theta, alpha and contralesional connectivity of beta at pre-training not only can discriminate between participants with good and poor recovery in the NG-AO group but also have significant correlations with post-training motor improvement, indicating their potential as predictive biomarkers of intervention-induced motor improvement.

### Brain connectivity for intervention prognosis

**Figure 6:**
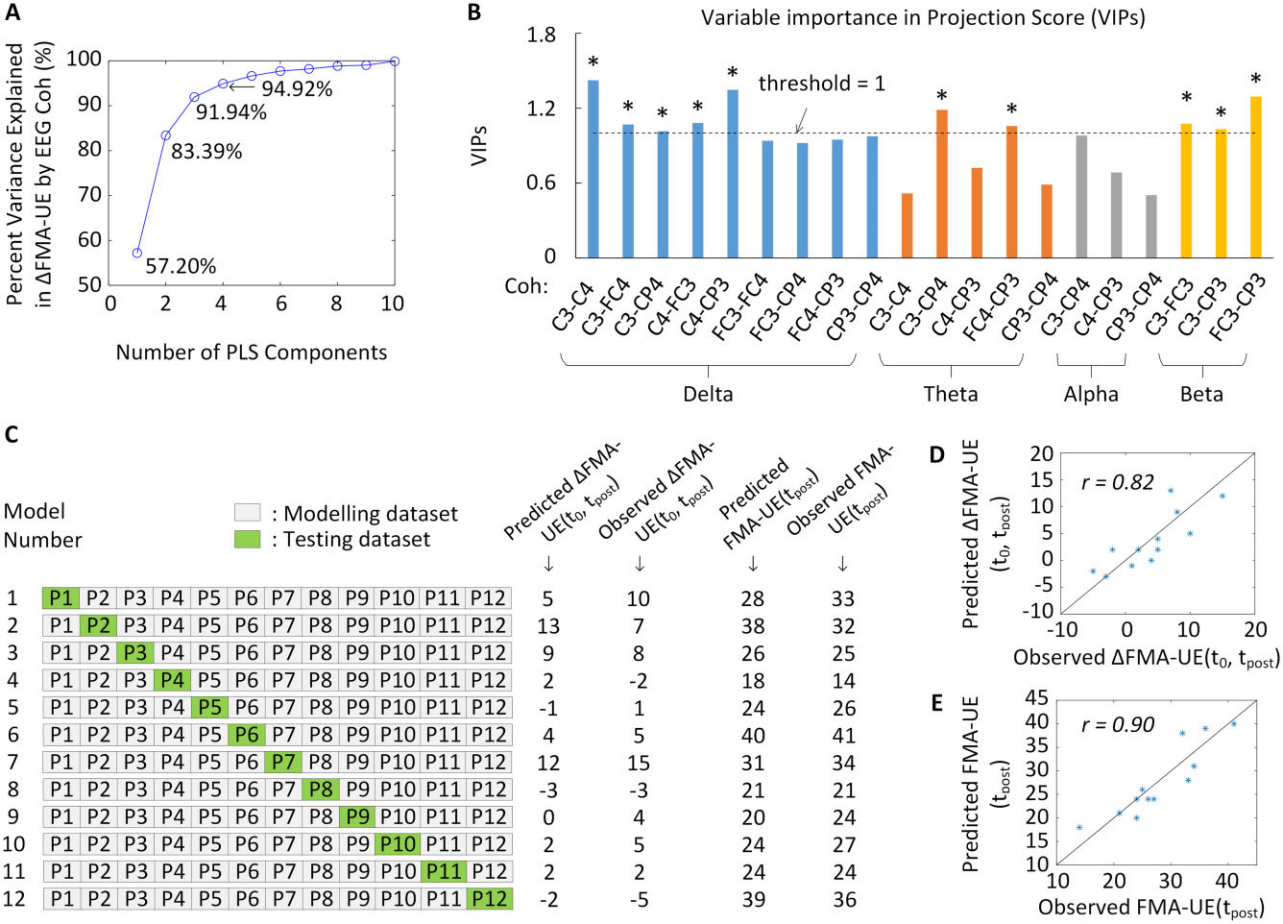
**Coherence-based biomarker for predicting intervention gains in participants in the NG-AO group.** (**A**) The change in percentage variance explained in intervention gains by EEG coherence with the increase in PLS components. Three components are needed to achieve more than 90% of the variance explained in intervention gains. (**B**) Variable importance in projection score for recovery-related EEG coherences. Nine pre-training coherences (delta: C3-C4, C3-FC4, C3-CP4, C4-FC3, C4-CP3; theta: C3-CP4, FC4-CP3; and beta: C3-FC3, C3-CP3, FC3-CP3) belonging to interhemispheric and contralesional local connectivity were selected as biomarkers for predicting intervention gains. (**C**) Leave-one-out cross-validation algorithm was used to predict ΔFMA-UE(t_0_, t_post_) and FMA-UE(t_post_) for each participant (P1, P2, …, P12) by establishing regression model with 11 observations for model training and 1 observation left for model testing. The grey block indicates the datasets for modelling and the green block indicates the datasets for testing. (**D**) The significant correlation between the predicted ΔFMA-UE(t_0_, t_post_) and observed ΔFMA-UE(t_0_, t_post_) (r=0.82) and (**E**) between the predicted FMA-UE(t_post_) and observed FMA-UE(t_post_) (r=0.90).

To further explore the relationship between brain functional connectivity and intervention gains in NG-AO group, PLS was applied with motor improvement-related EEG coherences as independent variables and ΔFMA-UE(t_0_, t_post_) as dependent variables. The fitted PLS model shows that 3 components were required to explain 90% of variance in the dependent variable, as shown in Fig. 6A. In the fitted PLS model, the variable importance in the projection score estimates the importance of each variable in the projection used in a PLS model.^47^ There is no consensus about the cut-off threshold on variable importance in the projection scores for variable selection, and a proper threshold between 0.83 and 1.21 can yield more relevant variables according to the performance of some variable selection methods when multicollinearity is present.^48^ In this study, Fig. 6B demonstrates the importance of each recovery-related pre-training resting EEG coherences for predicting intervention gains, and a relatively prevalent cut-off (1.0) was applied as the threshold for predictive biomarker selection.^47^ Interhemispheric delta (C3-C4, C3-FC4, C3-CP4, C4-FC3, C4-CP3), and theta (C3-CP4 and FC4-CP3) coherence and contralesional beta (C3-FC3, C3-CP3, FC3-CP3) coherence were selected as intervention predictive biomarker. Leave-one-out cross-validation was used to estimate the prediction error of the PLS model. Since there are 12 participants in NG-AO group, 12 regression models can be established with 11 observations for model training and 1 observation left for model testing in each model (Fig. 6C). The predicted, observed ΔFMA-UE(t_0_, t_post_) and predicted, observed FMA-UE(t_post_) for each participant (P1, P2, …, P12) are also listed in Fig. 6C. The coefficient of each biomarker in the 12 regression models can be refer to Supplementary Table 5. The results show that the fitted model is accurate, with a strong correlation between the predicted ΔFMA-UE(t_0_, t_post_) and observed ΔFMA-UE (t_0_, t_post_) (Fig. 6D; r=0.82) and between the predicted FMA-UE(t_post_) and observed FMA-UE(t_post_) (Fig. 6E; r=0.90). The root mean square error (RMSE) of prediction is 3.24 across all participants. The PLS regression analysis was not applied to nNG-text group since pre-training EEG coherences from participants in nNG-text group neither have a strong correlation with intervention gain (Fig. 5B and D) nor be able to discriminate between good and poor recovery participants (Fig. 3B, D, F and H).

## Discussion

This work provides an EEG-based brain connectivity biomarker for potentially predicting intervention gains of chronic strokes in a neural guided action observation training. By evaluating combined Interhemispheric delta (C3-C4, C3-FC4, C3-CP4, C4-FC3, C4-CP3), theta (C3-CP4 and FC4-CP3) coherence and Contralesional beta (C3-FC3, C3-CP3, FC3-CP3) coherence of EEG signal at pre-training, we can accurately predict the intervention gain for each participant in the NG-AO group. However, the results show that it cannot be applied in the nNG-text group indicating the brain connectivity biomarker is intervention specific for NG-AO training. This study’s findings can help stroke patients establish reasonable expectations and provide a basis for selecting candidates who are more likely to be benefitted from the NG-AO training.

### Intervention-specific prognosis

Most previous investigations of predictive biomarkers for stroke rehabilitation have been conducted with stroke patients in acute or subacute phases. For example, an ipsilesional loss of power in the alpha frequency band and an increase in the delta frequency band detected within 2 weeks of stroke has been linked to a poor outcome.^49^ Coherence in the beta frequency band between the ipsilesional M1 and the rest of the cortex had a positive linear relationship with upper-limb motor improvement during the first 3 months after stroke.^50^ Few studies have investigated predictive biomarkers after the chronic phase since spontaneous motor recovery tends to be slow during this period. At this time point, rehabilitation intervention could help chronic stroke patients show ongoing motor function improvements, making intervention-specific prognosis an essential issue for selecting candidates who are more likely to be benefitted. Several EEG-related intervention biomarkers have been discussed in previous studies.^6^^,^^30^^,^^51^ With these biomarkers, connectivity-based analyses of neuroimaging data allowed new insights into the pathophysiology underlying stroke-induced deficits, as they provided an *in vivo* systems-level perspective of the specific outcomes that a lesion has on neural networks.^26^ This study demonstrated that the EEG coherence network is informative in the chronic stage, pointing to its potential use as a predictive biomarker for a robot-assisted training combined with a neural guided strategy. In the NG-AO group, which involved neural guided strategy, the combination of interhemispheric delta and theta connectivity and contralesional beta connectivity at pre-training precisely predicted the intervention gains shown at the post-training assessment with a small prediction error (RMSE = 3.24) and a strong correlation between the predicted ΔFMA-UE(t_0_, t_post_) and observed ΔFMA-UE (t_0_, t_post_) (Fig. 6D; r=0.82) and between the predicted FMA-UE(t_post_) and observed FMA-UE(t_post_) (Fig. 6E; r=0.90). Among them, contralesional alpha connectivity was also significantly correlated with intervention gains at the 6-month follow-up, indicating its sensitivity to long-term motor improvements (r=-0.614, P<0.05). For the nNG-text group, in which participants underwent non-neural guided training, no brain connectivity had significant predictive effects for intervention gains (all P>0.05). In summary, brain connectivity networks may be sensitive for predicting closed-loop training effects, such as neural guided training, since closed-loop learning, in which online feedback of neural activation is provided to the participant for self-regulation, tends to affect behaviourally relevant functional network reorganization.^52–56^ Besides, this study also indicated that predictive biomarkers for one intervention may not applicable for another type of intervention.

### Interhemispheric and contralesional functional connectivity for predicting recovery

Connectivity-based approaches provide great insight into network reorganization in the acute and chronic phases after stroke and contribute to improving prognostic abilities and the development of therapeutic interventions, as discussed in many fMRI and EEG studies.^15^^,^^20^^,^^21^^,^^25–29^^,^^36^^,^^57–59^ As shown in Supplementary Figs. 1–4, interhemispheric and contralesional functional connectivity at pre-training had a significant correlation with motor improvement in the training group with neural guidance. Contralesional functional connectivity has been proven to be a useful biomarker related to motor impairment and recovery after stroke in a previous EEG study; i.e. Riahi et al.^60^ reported a negative regression coefficient associated with higher contralesional functional connectivity between motor areas and FMA scores, which is consistent with our research results. Dubovik et al.^61^ and Westlak et al.^34^ also reported a negative relationship between functional connectivity of contralesional areas and motor performance. With fMRI, a consistent finding has been a reduction in interhemispheric functional connectivity between cortical sensory and motor regions that correlates with sensorimotor dysfunction;^20^^,^^24^^,^^29^^,^^31^^,^^57^ e.g. Carter et al.^20^ found that interhemispheric functional connectivity indicating disruption of the somatomotor network had a significant positive correlation with upper-extremity impairments. Van Meer et al.^57^ showed that restoration of resting interhemispheric functional connectivity positively correlated with recovery of sensorimotor function. Puig et al.^31^ reported that stroke patients with good recovery outcomes had greater interhemispheric functional connectivity than patients with poor outcomes in a resting-state fMRI study. In this study, we also found a significant relationship between interhemispheric EEG coherence at pre-training and intervention gains after neural guided training (Supplementary Figs. 1–4). Interestingly, the negative relationship seemed to contrast with the above-mentioned findings from the results from resting fMRI. These contrasting results between EEG and fMRI, which were also reported in Dijkhuizen et al.,^23^ may be caused by different experimental setups, analysis algorithms or participants’ stroke periods. The more likely possibility may lie in the different imaging mechanisms of EEG (electrophysiological activity) and fMRI (cerebral blood flow). It requires further research to resolve the underlying methodological or biological causes of dissimilarities between fMRI- and EEG-based connectivity measurements. In contrast to previous research, this was the first study to apply the EEG-based brain connectivity network for intervention-specific prognosis for chronic stroke. The recovery of motor function after stroke is not only related to the location and volume of the damaged tissue but also related to the neural pathways affected by the damaged tissue.

It worth to note that there are two pathways significantly correlate to interventional gains [ΔFMA-UE(t_0_, t_post_)] in NG-AO group. The first one is between ipsilesional motor/motor connected cortex (including PM: FC4, M1: C4 and SI: CP4) and contralesional motor/motor connected cortex (including FC3, C3 and CP3), it may be served by abundant white-matter fibres in the human corpus callosum. The second one is in contralesional motor/motor connected cortex (among FC3, C3 and CP3) which may be served by local neural circuits. However, we tend not to make strong conclusions about the interpretation at anatomical level due to poor spatial resolution of EEG signal. Although it is difficult to precisely speculate motor improvement related internal neural pathways from EEG due to its low spatial resolution, it can still be inferred that the integrity of the interhemispheric and contralesional brain connectivity network plays an essential role in recovery during rehabilitation training. This may be because the contralesional network partially compensates for the function of the lesion-induced disruption of neural networks in ipsilesional hemisphere.

### Significant coherence frequency band

The results of the PLS regression (Fig. 6C) showed that the interhemispheric delta coherences (C3-C4, CP3-C4) and the contralesional beta coherence (FC3-CP3) were the top 3 contributors for predicting intervention gains in the NG-AO group. Recent literature has shown that cortical connectivity measured by the small world index in these two frequencies is related to motor impairments^32^^,^^62^^,^^63^ and recovery^33^ in acute stroke patients, which is consistent with our results although our data were collected from participants in the chronic phase. However, alpha connectivity has also been reported as a biomarker of network function that is linearly associated with motor performance in other studies.^61^ Also, resting delta and alpha coherence was found to be significantly decreased after motor imagery training.^64^ Although beta coherence had a larger contribution index than alpha coherence, alpha coherence also had a significant correlation coefficient with intervention gains [Supplementary Table 3; Coh(FC3, CP3), r=-0.696, P<0.05] in our results. We guess that there might be two frequency bands in the brain network that strongly correlate with intervention gains. Delta is a widely agreed upon and robust relevant frequency, while the other may be located in the alpha and beta range and perhaps slightly varies from person to person. The two different frequency bands may be responsible for conveying different kinds of information.

### The utility of EEG coherence for clinical application

The potential for translating EEG biomarkers into clinical practice remains positive because EEG has been widely used in medical research with the advantage of offering high-resolution temporal information and became a standard practice nowadays. Besides, the method of this study is straightforward, since the resting tasks can be performed easily, and the EEG coherence is easy to be calculated. More importantly, there is no discomfort for the participants. The EEG coherence network has clinical potential for predicting the effectiveness of neural guided interventions. It could also be utilized to select suitable candidates for NG-AO intervention.

### Limitation and future work

Firstly, the small sample size might be a limitation of this study. The main reason for raising this issue is the length of rehabilitation training which was comparatively long (2–3 months per participant). However, compared with the other published studies^6^^,^^65^ in the same field, the sample size of this study would be acceptable. Another round of recruitment and experiments might be needed to further validate the findings of this study. Secondly, the participants’ genders were imbalanced (18 out of 22 participants are male). Thus, the applicability of the findings of this study on female might be questionable. Furthermore, prior work^66^ shows that patients with a functionally intact corticospinal tract experience a better recovery of upper limb function at the sub-acute stage, and a better response to further treatment at the chronic stage. Not knowing the motor evoked potentials of patients in this study is another limitation.

Future work will be focussed on applying this study in the clinical training, e.g. the criteria for selecting suitable patients for NG-AO training. A connectivity threshold as introduced in similar research in Hordacre et al.^67^ may be applied to select chronic stroke participants who are likely to respond based on the predicted intervention gains which may benefit therapists and stroke participants by providing information for selecting participants before conducting the intervention. Besides, the influence of patients’ handedness laterality on the prediction accuracy of intervention gain can also be considered in future work.

## Supplementary material

Supplementary material is available at *Brain Communications* online.

## Supplementary Material

fcab214_Supplementary_Data

## Abbreviations

Coh =: coherence
ContraLHemi =: contralesional local connectivity
ContraL-SMA =: contralesional to SMA connectivity
FMA-UE =: Fugl-Meyer Assessment-Upper Extremity
InterHemi =: interhemispheric connectivity
IpsiLHemi =: ipsilesional local connectivity
IpsiL-SMA =: ipsilesional to SMA connectivity
Isch =: ischaemic
M1 =: primary motor cortex
NG-AO =: Neural Guided-Action Observation
nNG-text =: non-Neural Guided-text
PLS =: partial least squares
PM =: premotor cortex
SI =: somatosensory cortex
SMA =: supplementary motor area
