# Supplementary material for: Functional brain networks assessed with surface electroencephalography for predicting motor recovery in a neural guided intervention for chronic stroke
Source: Brain Commun. 2021 Sep 25;3(4):fcab214. doi: 10.1093/braincomms/fcab214 (PMC8936428; doi:10.1093/braincomms/fcab214)
Supplement: fcab214_Supplementary_Data [file fcab214_Supplementary_Data.docx]

**Supplementary Material**

Functional Brain Networks Assessed with Surface Electroencephalography for Predicting Motor Recovery in a Neural Guided Intervention for Chronic Stroke

Rui Sun^1^, Wan-wa Wong^2^, Jing Wang^3^, Xin Wang^4^, Raymond K. Y. Tong^4,^[[1]](#footnote-1)^*^

^1^ The Laboratory of Neuroscience for Education, Faculty of Education, the University of Hong Kong, Pokfulam, Hong Kong, China

^2^ Department of Psychiatry and biobehavioral sciences, David Geffen School of Medicine, University of California Los Angeles, Los Angeles, United States

^3^ School of Mechanical Engineering, Xi’an Jiaotong University, Shaanxi, China

^4^ Department of Biomedical Engineering, The Chinese University of Hong Kong, Shatin, Hong Kong, China

**BCI based robot-assisted training platform**

A BCI-based robot-assisted training platform was developed in Sun et. al.^1^ as shown in **Figure**[**1A**](https://www.frontiersin.org/articles/10.3389/fnhum.2017.00444/full#F1). The EEG signals were captured by 16 active electrodes (g.LADYbird, g.Tec Medical Engineering GmbH, Austria) and amplified by an amplifier (g.USBamp, g.Tec Medical Engineering GmbH, Austria), and then processed in a computer. A paradigm can be played in a fixed sequence to guide the subject to complete the training. A robotic hand^2^ was used to assist the paretic hand to grasp/open. It can be triggered either based on the result of the mu suppression algorithm that was calculated from the EEG signals or base on a random signal. The system can display a mu suppression score as feedback for guiding the subject to adjust their practice of motor imagery. EEG signals and time-markers were recorded for further analysis after all tasks were completed.

Mu suppression is believed to be associated with the activation of Mirror Neural System (MNS)^3^. To compute mu suppression, C3 or C4 was selected according to the structural MRI information of subject's ipsilesional hemisphere. The EEG data were converted to the frequency domain by a fast Fourier transform algorithm with a Hanning window covering the EEG data during the stimulus period or baseline period. The mean power in the mu band (8–13 Hz) for the selected electrode was calculated. The value of mu suppression score^4, 5^ can be calculated by the following equation:

$$MuSC=-\frac{{MuP}_{stimulus}-{MuP}_{baseline}}{{MuP}_{baseline}}*100$$

where $MuSC$ represents the Mu suppression score, ${MuP}_{stimulus}$ represents the mu power of EEG during stimulus period while ${MuP}_{baseline}$ represents the mu power of EEG during resting period collected before each training session.

**Intervention Procedure:**

During each intervention session, subject was required to sit in a height-adjustable chair with his/her: (1) shoulder positioned at 90° abduction; (2) elbow flexed at 90°; (3) arm pronated, such that the palm was directed medially; and (4) wrist positioned neutrally without any flexion/extension, as shown in **Figure**[**1B**](https://www.frontiersin.org/articles/10.3389/fnhum.2017.00444/full#F1). A cushion was used to support and maintain the position of the subject's arm. 100 repetitive trials were performed by each subject with intermittent rest after every 10 trials.

*Neural Guided-Action Observation Training*:  each trial began with dark screen for 2 s, followed by a fixation cross “+” displayed at the center of the screen for 2 s. Then, subjects in this group were asked to observe a video demonstrating either grasping or releasing a cup using the subjects' unaffected hand, and the video frames were flipped to pretend that the subjects were observing their affected hand to do those hand actions. Action observation and motor imagery during playback of the video with real-time EEG guidance to trigger the robot hand. The Robot hand was triggered to help hand open or grasp if mu suppression calculated from real-time EEG signals was above 20. Such score means that the ratio of the responding mu power between stimulus period and baseline period was below 80% according to the average results reported in Perry's study^6^.

*non-Neural Guided-Text Training*: each trial began with dark screen for 2 s, followed by a fixation cross “+” displayed at the center of the screen for 2 s. Then, the subjects in this group were instructed to imagine their affected hand movements during a text cue of showing “hand open” or “hand grasp”. Motor imagery during display of text instruction of movement without EEG guidance. Robot hand was randomly triggered regardless of the subjects' EEG signals. In order to maintain the two groups having comparable training intensity for the paretic hand, the “success rate” of triggering the robot hand in sham group was set as 80%, which was the similar level as that in neural guided group from our preliminary study.

All subjects were instructed to imagine the same movement with the affected hand during the video or text display. The display of experimental sequences for the two training paradigms was controlled by the Psychophysics Toolbox 3.0 (<http://psychtoolbox.org/>).

**Supplementary Figure 1** Larger degree of Interhemispheric Delta coherences in 9 pair of electrodes (C3-C4, C3-FC4, C3-CP4, FC3-C4, CP3-C4, FC3-FC4, FC3-CP4, CP3-FC4, CP3-CP4) have significant association with smaller ΔFMA-UE(t_0_, t_post_) in NG-AO group.


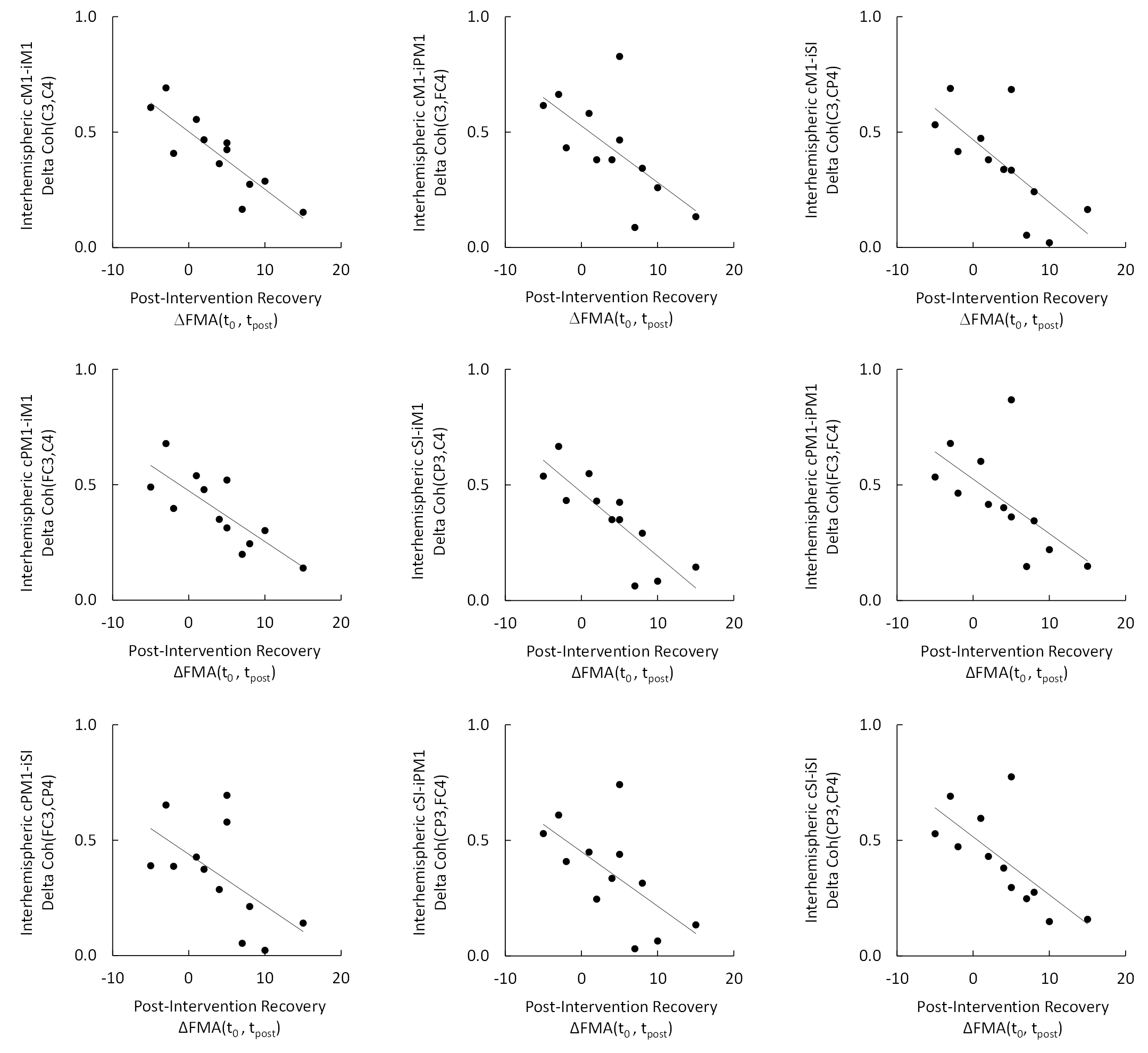


**Supplementary Figure 2** Larger degree of Interhemispheric Theta coherences in 5 pair of electrodes (C3-C4, C3-CP4, CP3-C4, CP3-FC4, CP3-CP4) and Contralesional Theta coherence in 1 pairs of electrodes (C3-CP3) have significant association with smaller ΔFMA-UE(t_0_, t_post_) in NG-AO group.

**
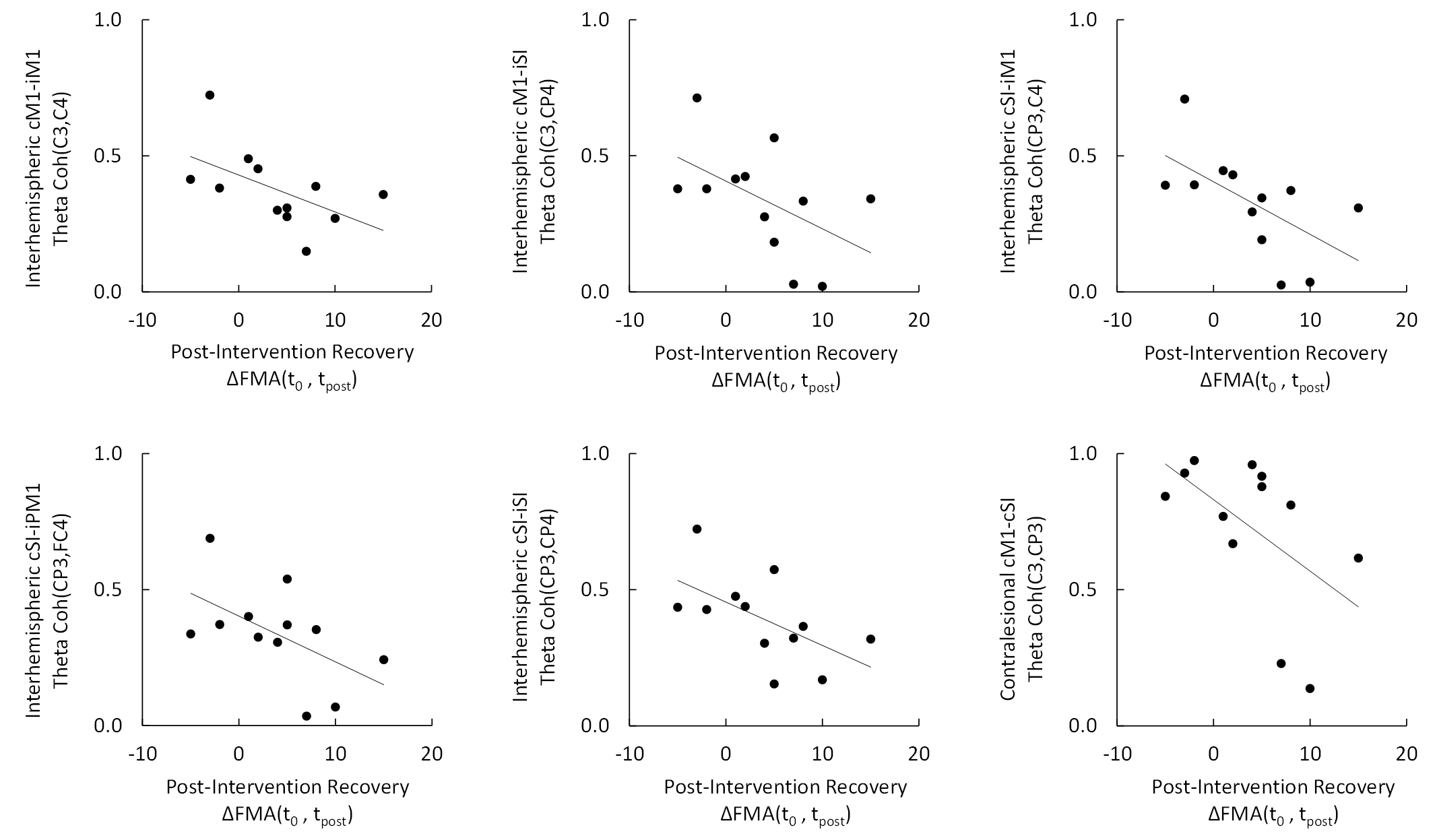
**

**Supplementary Figure 3 (A)** Larger degree of Interhemispheric Alpha coherences in 3 pair of electrodes (C3-CP4, CP3-C4, CP3-CP4) and Contralesional Alpha coherence in 3 pairs of electrodes (C3-FC3, FC3-CP3, C3-CP3) have significant association with smaller ΔFMA-UE(t_0_, t_post_) in NG-AO group. **(B)** Larger degree of Interhemispheric Alpha coherence in one pair of electrodes (FC3-CP3) have significant association with ΔFMA-UE(t_0_, t_6M_) in NG-AO group.


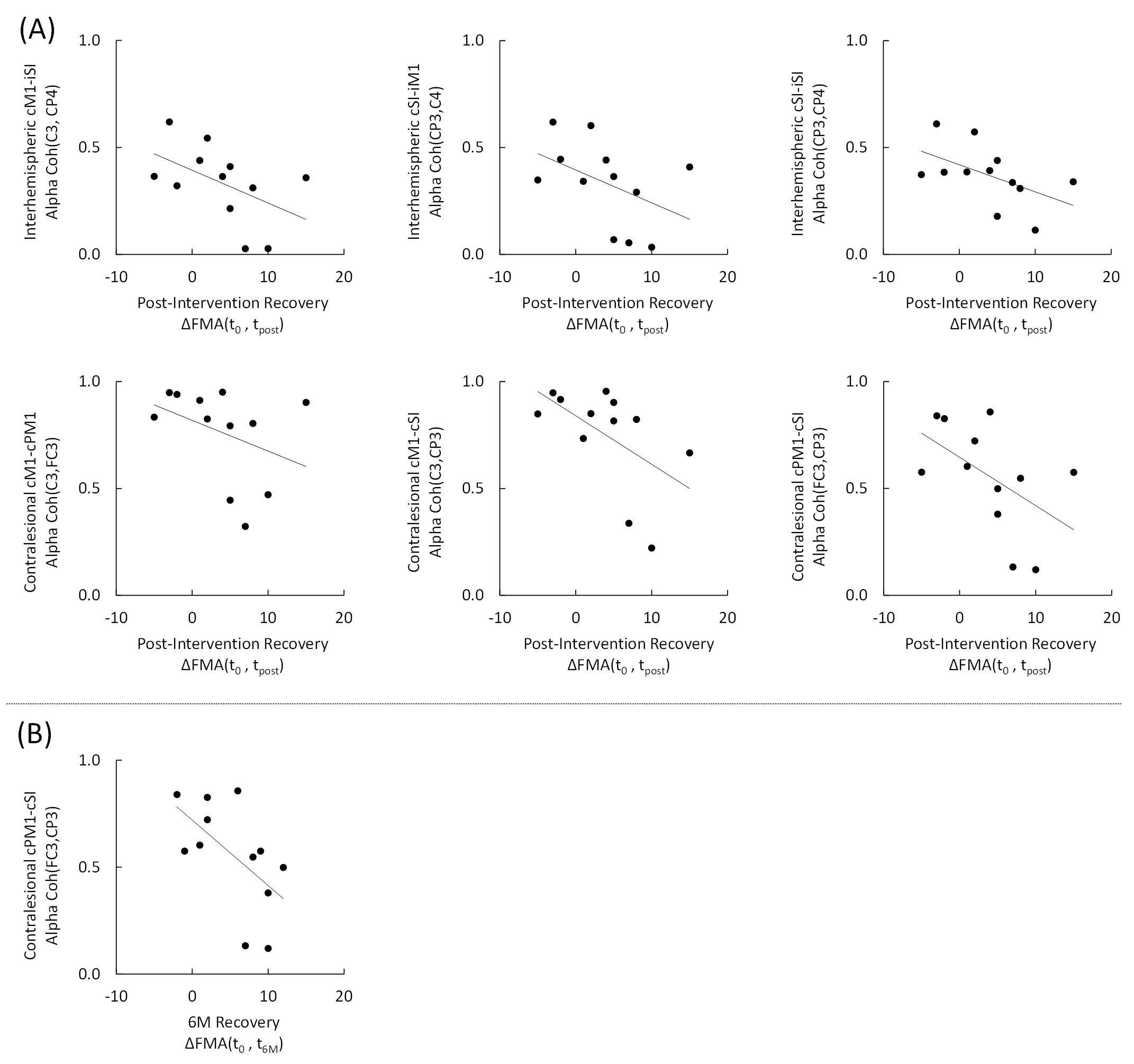


**Supplementary Figure 4** Larger degree of Contralesional Beta coherence in 3 pairs of electrodes (C3-FC3, FC3-CP3, C3-CP3) have significant association with smaller ΔFMA-UE(t_0_, t_post_) in NG-AO group.


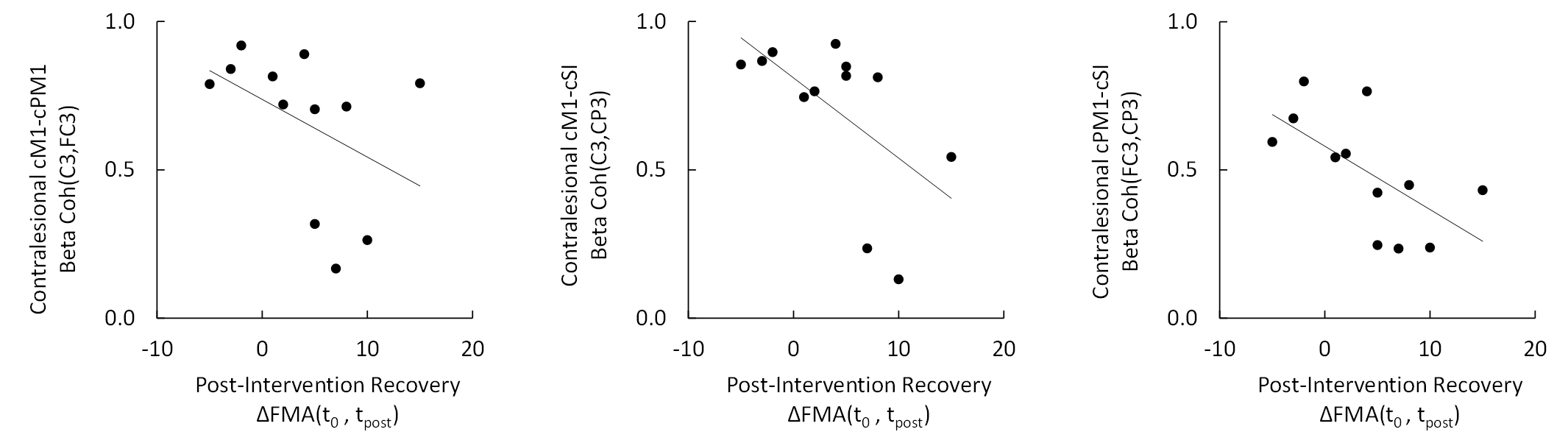


**Supplementary Table 1** Correlation Coefficients between Pre-intervention EEG Delta Coherences and ΔFMA-UE at Post-intervention and at 6-month Follow-up in both groups

| **Delta Coh** | **NG-AO Group** | | **nNG-text Group** | |
| --- | --- | --- | --- | --- |
|  | ΔFMA-UE  (t_0_, t_post_) | ΔFMA-UE  (t_0_, t_6M_) | ΔFMA-UE  (t_0_, t_post_) | ΔFMA-UE  (t_0_, t_6M_) |
| Coh (C3, C4) | **-0.834**** | -0.353 | -0.234 | 0.128 |
| Coh (C3, FC4) | **-0.702*** | -0.107 | -0.226 | 0.051 |
| Coh (C3, CP4) | **-0.840**** | -0.397 | 0.075 | 0.204 |
| Coh (C4, FC3) | **-0.801**** | -0.309 | -0.050 | 0.119 |
| Coh (C4, CP3) | **-0.901**** | -0.532 | -0.151 | 0.255 |
| Coh (FC3, FC4) | **-0.768*** | -0.259 | -0.117 | 0.043 |
| Coh (FC3, CP4) | **-0.663*** | -0.201 | 0.042 | 0.221 |
| Coh (FC4, CP3) | **-0.685*** | -0.154 | -0.025 | -0.009 |
| Coh (CP3, CP4) | **-0.807**** | -0.399 | 0.218 | 0.468 |
| Coh (C3, FC3) | -0.398 | -0.174 | 0.059 | 0.434 |
| Coh (C3, CP3) | -0.486 | -0.066 | -0.008 | 0.149 |
| Coh (FC3, CP3) | -0.238 | 0.107 | -0.276 | -0.136 |
| Coh (C4, FC4) | -0.552 | -0.322 | 0.552 | 0.417 |
| Coh (C4, CP4) | -0.492 | -0.496 | 0.385 | 0.451 |
| Coh (FC4, CP4) | -0.522 | -0.328 | 0.552 | 0.417 |
| Coh (C3, Cz) | -0.156 | 0.342 | -0.343 | 0.187 |
| Coh (FC3, Cz) | -0.266 | 0.155 | -0.033 | -0.017 |
| Coh (CP3, Cz) | -0.211 | 0.401 | -0.192 | 0.392 |
| Coh (C4, Cz) | **-0.798**** | -0.369 | -0.025 | 0.068 |
| Coh (FC4, Cz) | -0.514 | 0.105 | 0.142 | 0.255 |
| Coh (CP4, Cz) | -0.606 | -0.118 | 0.192 | 0.323 |

**Supplementary Table 2** Correlation Coefficients between Pre-intervention EEG Theta Coherences and ΔFMA-UE at Post-intervention and at 6-month Follow-up in both groups

| **Theta Coh** | **NG-AO Group** | | **nNG-text Group** | |
| --- | --- | --- | --- | --- |
|  | ΔFMA-UE  (t_0_, t_post_) | ΔFMA-UE  (t_0_, t_6M_) | ΔFMA-UE  (t_0_, t_post_) | ΔFMA-UE  (t_0_, t_6M_) |
| Coh (C3, C4) | **-0.608*** | -0.369 | -0.109 | 0.051 |
| Coh (C3, FC4) | -0.514 | -0.085 | 0.067 | -0.145 |
| Coh (C3, CP4) | **-0.608*** | -0.251 | 0.033 | 0.247 |
| Coh (C4, FC3) | -0.514 | -0.218 | -0.025 | 0.077 |
| Coh (C4, CP3) | **-0.702**** | -0.479 | -0.050 | 0.289 |
| Coh (FC3, FC4) | -0.453 | -0.063 | -0.084 | 0.128 |
| Coh (FC3, CP4) | -0.398 | 0.039 | 0.201 | 0.298 |
| Coh (FC4, CP3) | **-0.575*** | -0.096 | 0.017 | 0.017 |
| Coh (CP3, CP4) | **-0.586*** | -0.322 | 0.075 | 0.545 |
| Coh (C3, FC3) | -0.503 | -0.322 | -0.184 | 0.162 |
| Coh (C3, CP3) | **-0.608*** | -0.157 | -0.100 | 0.064 |
| Coh (FC3, CP3) | -0.409 | -0.047 | -0.393 | -0.034 |
| Coh (C4, FC4) | -0.530 | -0.380 | 0.234 | 0.230 |
| Coh (C4, CP4) | -0.298 | -0.399 | 0.234 | 0.366 |
| Coh (FC4, CP4) | -0.431 | -0.207 | 0.377 | 0.281 |
| Coh (C3, Cz) | -0.220 | 0.351 | -0.385 | 0.094 |
| Coh (FC3, Cz) | -0.193 | 0.109 | -0.218 | 0.221 |
| Coh (CP3, Cz) | -0.275 | 0.301 | -0.117 | 0.426 |
| Coh (C4, Cz) | -0.440 | -0.415 | 0.050 | 0.162 |
| Coh (FC4, Cz) | -0.321 | 0.173 | 0.276 | 0.272 |
| Coh (CP4, Cz) | -0.275 | -0.005 | 0.092 | 0.238 |

**Supplementary Table 3** Correlation Coefficients between Pre-intervention EEG Alpha Coherences and ΔFMA-UE at Post-intervention and at 6-month Follow-up in both groups

| **Alpha Coh** | **NG-AO Group** | | **nNG-text Group** | |
| --- | --- | --- | --- | --- |
|  | ΔFMA-UE  (t_0_, t_post_) | ΔFMA-UE  (t_0_, t_6M_) | ΔFMA-UE  (t_0_, t_post_) | ΔFMA-UE  (t_0_, t_6M_) |
| Coh (C3, C4) | -0.525 | -0.449 | -0.075 | 0.102 |
| Coh (C3, FC4) | -0.481 | -0.132 | 0.218 | 0.162 |
| Coh (C3, CP4) | **-0.641*** | -0.331 | 0.159 | 0.153 |
| Coh (C4, FC3) | -0.464 | -0.366 | -0.209 | 0.068 |
| Coh (C4, CP3) | **-0.569*** | -0.397 | -0.192 | 0.264 |
| Coh (FC3, FC4) | -0.287 | -0.124 | 0.226 | 0.570 |
| Coh (FC3, CP4) | -0.409 | -0.157 | 0.084 | 0.196 |
| Coh (FC4, CP3) | -0.508 | -0.187 | -0.192 | -0.017 |
| Coh (CP3, CP4) | **-0.619*** | -0.317 | 0.109 | 0.630 |
| Coh (C3, FC3) | **-0.564*** | -0.444 | 0.167 | -0.009 |
| Coh (C3, CP3) | **-0.669*** | -0.278 | 0.067 | -0.047 |
| Coh (FC3, CP3) | **-0.696*** | **-0.614*** | -0.243 | -0.085 |
| Coh (C4, FC4) | -0.552 | -0.438 | 0.402 | 0.213 |
| Coh (C4, CP4) | -0.282 | -0.408 | 0.318 | 0.196 |
| Coh (FC4, CP4) | -0.508 | -0.358 | 0.452 | 0.196 |
| Coh (C3, Cz) | -0.156 | 0.114 | 0.067 | 0.153 |
| Coh (FC3, Cz) | -0.147 | -0.073 | -0.184 | 0.026 |
| Coh (CP3, Cz) | -0.138 | 0.282 | -0.134 | 0.306 |
| Coh (C4, Cz) | -0.422 | -0.346 | 0.259 | 0.221 |
| Coh (FC4, Cz) | -0.422 | -0.159 | 0.393 | 0.264 |
| Coh (CP4, Cz) | -0.367 | -0.333 | 0.100 | -0.043 |

**Supplementary Table 4** Correlation Coefficients between Pre-intervention EEG Beta Coherences and ΔFMA-UE at Post-intervention and at 6-month Follow-up in both groups

| **Beta Coh** | **NG-AO Group** | | **nNG-text Group** | |
| --- | --- | --- | --- | --- |
|  | ΔFMA-UE  (t_0_, t_post_) | ΔFMA-UE  (t_0_, t_6M_) | ΔFMA-UE  (t_0_, t_post_) | ΔFMA-UE  (t_0_, t_6M_) |
| Coh (C3, C4) | -0.464 | -0.231 | 0.469 | -0.238 |
| Coh (C3, FC4) | -0.298 | 0.036 | 0.285 | -0.043 |
| Coh (C3, CP4) | -0.541 | -0.399 | 0.360 | -0.128 |
| Coh (C4, FC3) | -0.497 | -0.350 | 0.343 | -0.009 |
| Coh (C4, CP3) | -0.547 | -0.328 | 0.100 | 0.213 |
| Coh (FC3, FC4) | -0.304 | -0.091 | 0.444 | 0.332 |
| Coh (FC3, CP4) | -0.492 | -0.171 | 0.393 | 0.136 |
| Coh (FC4, CP3) | -0.519 | -0.176 | 0.017 | 0.009 |
| Coh (CP3, CP4) | -0.442 | -0.421 | 0.251 | 0.468 |
| Coh (C3, FC3) | **-0.597*** | -0.433 | 0.092 | 0.034 |
| Coh (C3, CP3) | **-0.691*** | -0.237 | -0.042 | 0.140 |
| Coh (FC3, CP3) | **-0.729**** | -0.499 | 0.151 | 0.247 |
| Coh (C4, FC4) | -0.503 | -0.328 | 0.418 | 0.468 |
| Coh (C4, CP4) | -0.359 | -0.386 | 0.393 | 0.136 |
| Coh (FC4, CP4) | -0.298 | -0.259 | 0.477 | 0.230 |
| Coh (C3, Cz) | 0.092 | 0.542 | 0.360 | -0.077 |
| Coh (FC3, Cz) | 0.018 | 0.337 | 0.025 | 0.009 |
| Coh (CP3, Cz) | -0.422 | 0.128 | -0.084 | 0.136 |
| Coh (C4, Cz) | -0.046 | 0.128 | 0.427 | 0.009 |
| Coh (FC4, Cz) | 0.064 | 0.405 | 0.351 | 0.332 |
| Coh (CP4, Cz) | -0.018 | 0.087 | 0.351 | -0.128 |

**Supplementary Table 5** The coefficient of each biomarker in 12 PLS regression models established by leave-one-out cross-validation algorithm in Fig. 6

| Model | Delta | | | | | Theta | | Beta | | |
| --- | --- | --- | --- | --- | --- | --- | --- | --- | --- | --- |
|  | Coh  (C3,C4) | Coh  (C3,FC4) | Coh  (C3,CP4) | Coh  (FC3,C4) | Coh  (CP3,C4) | Coh  (C3,CP4) | Coh  (CP3,FC4) | Coh  (C3,FC3) | Coh  (C3,CP3) | Coh  (FC3,CP3) |
| 1 | -13.25 | -5.43 | -1.53 | -9.38 | -10.75 | 9.87 | 9.41 | 5.75 | 0.90 | -14.23 |
| 2 | -12.43 | -7.48 | -0.46 | -7.37 | -10.48 | 8.51 | 6.56 | 1.14 | -2.65 | -11.38 |
| 3 | -12.28 | -5.24 | -1.23 | -8.10 | -11.19 | 8.95 | 8.33 | 5.14 | -2.00 | -13.46 |
| 4 | -12.76 | -4.70 | -1.95 | -8.36 | -10.80 | 7.14 | 8.20 | 4.64 | -0.99 | -9.93 |
| 5 | -12.56 | -5.19 | -2.02 | -8.80 | -11.18 | 8.84 | 8.58 | 4.50 | -0.75 | -13.53 |
| 6 | -10.10 | -6.76 | -7.42 | -9.40 | -5.58 | 8.48 | 10.00 | 5.69 | -1.14 | -14.66 |
| 7 | -12.87 | -0.89 | -4.81 | -3.56 | -13.79 | 5.02 | 9.39 | 4.09 | 0.18 | -10.84 |
| 8 | -12.07 | -4.73 | -2.18 | -8.44 | -10.51 | 8.54 | 8.58 | 4.56 | -0.89 | -13.66 |
| 9 | -12.24 | -4.12 | -1.64 | -8.31 | -10.81 | 10.13 | 9.90 | 4.02 | -2.10 | -16.63 |
| 10 | -12.24 | -5.25 | -1.95 | -7.42 | -11.69 | 9.96 | 7.91 | 6.83 | -3.74 | -12.26 |
| 11 | -11.97 | -5.25 | -2.18 | -8.03 | -10.55 | 8.92 | 8.12 | 4.67 | -1.14 | -13.56 |
| 12 | -9.80 | -2.26 | -0.98 | -9.93 | -10.25 | 7.17 | 4.74 | 6.46 | -1.05 | -14.76 |
| Mean | -12.05 | -4.77 | -2.36 | -8.09 | -10.63 | 8.46 | 8.31 | 4.79 | -1.28 | -13.24 |
| Std | 1.04 | 1.77 | 1.91 | 1.62 | 1.85 | 1.44 | 1.48 | 1.46 | 1.23 | 1.87 |

**Reference**

1. Sun R, Wong WW, Wang J, Tong KY. Changes in electroencephalography complexity using a brain computer interface-motor observation training in chronic stroke patients: a fuzzy approximate entropy analysis. *Front Hum Neurosci*. 2017;11:444.

2. Tong KY, Pang MKP, Chen M, Ho SK, Zhou HF, Ng TWD, inventors; Wearable power assistive device for helping a user to move their hand. US 2013.

3. Bartur G, Pratt H, Dickstein R, Frenkel-Toledo S, Geva A, Soroker N. Electrophysiological manifestations of mirror visual feedback during manual movement. *Brain Res*. 2015;1606:113-124.

4. Oberman LM, Ramachandran VS, Pineda JA. Modulation of mu suppression in children with autism spectrum disorders in response to familiar or unfamiliar stimuli: the mirror neuron hypothesis. *Neuropsychologia*. 2008;46(5):1558-1565.

5. Braadbaart L, Williams JH, Waiter GD. Do mirror neuron areas mediate mu rhythm suppression during imitation and action observation? *Int J Psychophysiol*. 2013;89(1):99-105.

6. Perry A, Bentin S. Mirror activity in the human brain while observing hand movements: a comparison between EEG desynchronization in the mu-range and previous fMRI results. *Brain Res*. 2009;1282:126.

1. * Correspondence:

   Raymond K. Y. Tong

   Address: Office Rm 429, Ho Sin Hang Engineering Building, The Chinese University of Hong Kong, Shatin, N.T., Hong Kong

   Telephone number: +852 3943 8454

   Fax number: +852 2603 5558

   E-mail address: [kytong@cuhk.edu.hk](mailto:kytong@cuhk.edu.hk) [↑](#footnote-ref-1)
